# Ceruloplasmin Status in Medullary Thyroid Carcinoma

**Published:** 2020-01

**Authors:** Fatemeh RAZAVI, Sara SHEIKHOLESLAMI, Marjan ZARIF YEGANEH, Tahereh NAJI, Mehdi HEDAYATI

**Affiliations:** 1 Pharmaceutical Sciences Branch, Islamic Azad University, Tehran, Iran; 2 Chronic Respiratory Diseases Research Center, National Research Institute of Tuberculosis and Lung Diseases, Shahid Beheshti University of Medical Sciences, Tehran, Iran; 3 Cellular and Molecular Endocrine Research Center, Research Institute for Endocrine Sciences, Shahid Beheshti University of Medical Sciences, Tehran, Iran

## Dear Editor-in-Chief

Thyroid cancer is the most common malignancy of the endocrine system (1) with four histopathological forms of Papillary (PTC), Follicular (FTC), Medullary (MTC) and Anaplastic (ATC). Among all subtypes of thyroid cancers, medullary thyroid carcinoma is the most common cause of mortality and morbidity (2). Ceruloplasmin is a kind of adipokine, expressed in adipose tissue (3). The present study was done based on the hypothesis which some adipokines such as ceruloplasmin could be related to medullary thyroid carcinoma. Before the current study, we measured the serum Vaspin, Retinol Binding Protein-4 (4), adiponectin and leptin as well (5). Since some studies have revealed the association between the development of thyroid cancer and adipokines, this study aimed to investigate this relationship between medullary thyroid cancer and ceruloplasmin.

Ninety individuals referred to the Research Institute for endocrine sciences, Shahid Beheshti University of Medical sciences, Tehran, Iran, were enrolled in this study.

This study was done following guidelines approved by mentioned institution ethical committee. All participants signed informed consent.

This population consisted of 26 females and 19 males (mean age 33.0±10.7 yr) with Medullary Thyroid Carcinoma pathological diagnosis and exciting RET proto-oncogene mutations in exons 10, 11, 13–16. Moreover, we selected a healthy age, sex and BMI matched control group consisted of 25 females and 20 males (mean age 32.2±10.0 yr) without thyroid disorders and with normal thyroid function tests. If one who had any thyroid cancer symptoms and family historical reports or had any mutation in exons 10, 11, 13–16 were excluded from the study.

Ten ml of blood was collected from a vein of the left arm in tubes with EDTA and invert tubes 10 times gently and rested for 30 min at room temperature. Then centrifuged at 3000 rpm for 10 min and plasma was separated. The plasma samples were stored at −20 °C. Genomic DNA was extracted by the standard Salting Out/Proteinase K method and Mutations detection were performed through direct DNA sequencing (ABI 3100 Genetic Analyzer and Big Dye Terminator v3.1 Cycle Sequencing Kit ®, Applied Biosystems, California, USA). Ceruloplasmin plasma levels were measured from plasma using a human total Ceruloplasmin Sandwich type ELISA kit with sensitivity of 0.039 ng/ml and intra coefficient of variation of 6.4%.

Data were analyzed using SPSS statistical software (ver. 20 (Chicago, IL, USA). Demographic and biochemical characteristics of patients and control groups are shown in Table 1.

**Table 1:** BMI, age, ceruloplasmin level and statistical difference in patients and control groups

***Variable***	***Case***	***Control***	**P-*value***
No. of subjects	45	45	NS^**^
No. of thyroidectomy	23	-	NS
Age (yr)	33.0 ± 10.7	32.2 ± 10.0	NS
BMI (kg/m^2^)	26.2 ± 1.3	25.8 ± 10.1	NS
Ceruloplasmin (μg/ml)^*^	215.7 ± 80.3	222.7 ± 70.7	NS

* Data are presented as mean ± SD

** Not significant. *P*<0.05 is significant

Plasma ceruloplasmin levels were 215.7 ± 80.3 and 222.7 ±70.7 μg/ml in case and control groups respectively. No significant difference was seen between plasma levels of ceruloplasmin of patients and control groups (*P*=0.7). There was no significant difference in celruloplsmin concentration between case and control groups divided by gender. However, the ceruloplasmin level was slightly increased among male Medullary Thyroid Carcinoma patients (206.3 ± 78.3 μg/ml) than male control group (201.8 ± 50.3 μg/ml). But it was not significant.

To the best of our knowledge, this is the first study about plasma levels of ceruloplasmin among individuals with medullary thyroid carcinoma. Since we found no difference in the plasma levels of ceruloplasmin between two groups, currently it cannot be used as a diagnostic marker in order to distinguish patients with medullary thyroid carcinoma and healthy individuals.
